# Supplementary material for: Evaluation of the diagnostic and prognostic values of serum HSP90α in sepsis patients: a retrospective study
Source: PeerJ. 2022 Mar 10;10:e12997. doi: 10.7717/peerj.12997 (PMC8918145; doi:10.7717/peerj.12997)
Supplement: Supplemental Information 1 [file peerj-10-12997-s001.docx]

Supplementary Table 1 Univariate logistic regression analysis for sepsis.

| Univariate logistic  regression analysis | B | S. E. | Wald | *P*-value | Odds ratio | 95%CI |
| --- | --- | --- | --- | --- | --- | --- |
| HSP90α | 0.008 | 0.001 | 26.190 | <0.001 | 1.302 | 1.059-1.600 |
| SOFA | 0.212 | 0.051 | 17.307 | <0.001 | 1.236 | 1.119-1.366 |
| PCT | 0.014 | 0.006 | 5.099 | 0.024 | 1.014 | 1.002-1.026 |
| BUN | 0.045 | 0.019 | 5.371 | 0.020 | 1.046 | 1.007-1.087 |
| Crea | 0.001 | 0.002 | 4.628 | 0.031 | 1.002 | 1.000-1.004 |
